# Kinase GSK3β functions as a suppressor in colorectal carcinoma through the FTO‐mediated MZF1/c‐Myc axis

**DOI:** 10.1111/jcmm.16291

**Published:** 2021-02-02

**Authors:** Zeyan Zhang, Qianfu Gao, Shanchao Wang

**Affiliations:** ^1^ Anorectal Department Linyi People’ s Hospital Linyi China

**Keywords:** colorectal carcinoma, fat mass and obesity‐associated protein, glycogen synthase kinase 3 beta, myeloid zinc finger 1, *N*^6^‐methyladenosine

## Abstract

Colorectal carcinoma (CRC) poses heavy burden to human health and has an increasing incidence. Currently, the existing biomarkers for CRC bring about restrained clinical benefits. GSK3β is reported to be a novel therapeutic target for this disease but with undefined molecular mechanisms. Thus, we aimed to investigate the regulatory effect of GSK3β on CRC progression via FTO/MZF1/c‐Myc axis. Firstly, the expression patterns of GSK3β, FTO, MZF1 and c‐Myc were determined after sample collection. Lowly expressed GSK3β but highly expressed FTO, MZF1 and c‐Myc were found in CRC. After transfection of different overexpressed and interference plasmids, the underlying mechanisms concerning GSK3β in CRC cell functions were analysed. Additionally, the effect of GSK3β on FTO protein stability was assessed followed by detection of MZF1 m6A modification and MZF1‐FTO interaction. Mechanistically, GSK3β mediated ubiquitination of demethylase FTO to reduce FTO expression. Besides, GSK3β inhibited MZF1 expression by mediating FTO‐regulated m6A modification of MZF1 and then decreased the proto‐oncogene c‐Myc expression, thus hampering CRC cell proliferation. We also carried out in vivo experiment to verify the regulatory effect of GSK3β on CRC via FTO‐mediated MZF1/c‐Myc axis. It was found that GSK3β inhibited CRC growth in vivo which was reversed by overexpressing c‐Myc. Taken together, our findings indicate that GSK3β suppresses the progression of CRC through FTO‐regulated MZF1/c‐Myc axis, shedding light onto a new possible pathway by which GSK3β regulates CRC.

## 1 INTRODUCTION

Most of colorectal cancers (CRCs) are adenocarcinoma, which is a malignant tumour developing from glandular cells of the rectum and colon.^1^ CRC presents a high mortality and an increasing incidence and leads to about 900 000 cancer‐related deaths globally.^2^ Genetic and environmental factors both have a substantial effect on the aetiology of CRC, the former including Lynch syndrome and familial adenomatous polyposis and the latter consisting of smoking, obesity and lack of physical exercise.^3^ Anaemia, rectal bleeding, abdominal pain and bowel dysfunction are the major clinical symptoms of CRC patients, but CRC is often asymptomatic at the early stage,^4^ leading to difficulty in its diagnosis. Apart from chemotherapy regimens, novel targeted therapies such as phosphoinositide 3‐kinase (PI3K)‐targeted therapy may be effective in the treatment of patients with CRC^5^ and the glycogen synthase kinase 3 beta (GSK3β) mediates cell activities via the PI3K/AKT signalling pathway,^6^ implying the regulatory role of GSK3β in CRC. However, the molecular mechanisms underlying the CRC initiation still remain undefined and current targeted agents bring restrained clinical benefit, so more innovative therapies targeting CRC are expected to be found for treating this malignant neoplasm.

Previous study has shown that the kinase GSK3β is an inhibitor gene for CRC occurrence.^7^ Additionally, GSK3β could promote the ubiquitination of fat mass and obesity‐associated protein (FTO) by regulating FTO phosphorylation, thereby elevating FTO degradation and suppressing FTO protein expression.^8^ As a N6‐methyladenosine (m6A) demethylase, FTO could inhibit the m6A methylation of mRNA, thereby affecting cell viability.^9^ Moreover, FTO was found to be overexpressed in the cells of patients with gastric cancer,^10^ suggesting its oncogenic role. Specifically, FTO is reported to remove m6A mRNA methylation of myeloid zinc finger 1 (MZF1), thus increasing the expression of MZF1.^11^ MZF1 is a carcinogenic transcription factor that regulates the invasion of multiple solid cancers including CRC, cervical cancer and hepatocellular carcinoma, and it has also been implicated in PI3K pathway.^12^ Hence, we speculated that inhibition of MZF1 can serve as an efficient therapy to prevent CRC progression, and the kinase GSK3β may inhibit the expression of FTO and MZF1, thus functioning as a suppressor of CRC. To prove the hypothesis and explore the molecular mechanisms, we have performed a series of experiments.

## 2 MATERIALS AND METHODS

### 2.1 Ethics statement

All research protocols were conducted with approval of the Clinical Research Ethics Committee of Linyi People’ s Hospital and in line with the Declaration of Helsinki (Ethnical number: 201 805 014). Informed consent was signed by all patients and/or legal guardians before experiments. All animal experiments were performed according to the Animal Ethics Committee of Linyi People’ s Hospital (Ethnical number: 201 807 005). We have tried the best to minimize the number of animals used in the tests and their suffering.

### 2.2 Clinical sample collection

Colorectal carcinoma tissues and adjacent normal tissues were excised from 57 patients with CRC diagnosed in Linyi People’ s Hospital from August 2018 to August 2019 and stored in liquid nitrogen for subsequent research. None of these patients received radiation therapy or pre‐operative chemotherapy.

### 2.3 Cell culture and grouping

Colorectal carcinoma cells SW480 (ZQ0063), SW620 (LZQ0014) and HCT‐8 (ZQ0331) purchased from Shanghai Zhongqiaoxinzhou Biotech Co, Ltd. as well as immortalized normal human intestinal epithelial cell (HIEC) from American Type Culture Collection (ATCC) were incubated in Dulbecco's Modified Eagle Medium (D554;, Sigma‐Aldrich Chemical Company) supplemented with 10% foetal bovine serum, 100 ug/mL penicillin and 50 ug/mL streptomycin in a constant temperature incubator with 5% CO_2_ at 37°C. After that, the cells in adherent growth were detached with 0.25% trypsin (T4799, Sigma), followed by collection of the cells in logarithmic growth for subsequent experiments.

The mammalian cell vector pCMV6‐AC‐GFP (Hunan Fenghui Biotechnology Co, Ltd.) contains the sequence of the turbo green fluorescent protein (tGFP) at the ORF site, a variant of the ‘CopGFP’ from copepod Pontellina plumata (Dana, 1849) (Crustacea: Pontellidae). The ORF is behind a cytomegalovirus promoter that could overexpress the protein to be tested. The plasmid was amplified overnight through transforming competent DH5αE‐coli. Finally, positive clones were collected by adding 100 µg/mL ampicillin.^13^

The overexpressed plasmid pCMV6‐AC‐GFP and the interference plasmid pGPU6/Neo were purchased from Fenghuishengwu Co., Ltd. and GenePharma Co. Ltd., respectively. The cells were transfected with oe‐GSK3β, oe‐FTO, oe‐MZF1, sh‐FTO or sh‐c‐Myc according to the instructions of lipofectamine 2000 (Invitrogen) kit (11 668 019. Thermo fisher) and collected for subsequent experiments.

### 2.4 Reverse transcription quantitative polymerase chain reaction (RT‐qPCR)

Total RNA was isolated from tissues and cells using TRIzol (Invitrogen), and the concentration and purity of which were measured using a NanoDrop 2000 micro ultraviolet spectrophotometer (1011U; NanoDrop Technologies Inc.). The RNA was reversely transcribed into cDNA according to the instructions of PrimeScript reagent Kit (RR047A; Takara Holdings Inc.), and primers for MZF1 were designed and synthesized by Takara (Table 1). ABI7500 qPCR instrument (7500; Applied Biosystems) was employed for real‐time fluorescent qPCR detection. β‐actin was used as an internal reference, and the fold changes were calculated by means of relative quantification (2^‐△△Ct^ method).

**TABLE 1:** Primer sequences

	Sequences
MZF1	F: 5'‐TGCAGGTGAAAGAGGAGTCA‐3'
	R: 5'‐AGTCTTGCTGTGGGGAAAGA‐3'
β‐actin	F: 5'‐CATGTACGTTGCTATCCAGGC‐3'
	R: 5'‐CTCCTTAATGTCACGCACGAT‐3'

### 2.5 Western blot analysis

Total protein in tissues or cells was extracted with radio immunoprecipitation assay (RIPA) lysis buffer containing phenylmethanesulfonyl fluoride (P0013C; Beyotime Institute of Biotechnology), with the concentration detected by BCA kit. After sodium dodecyl sulphate‐polyacrylamide gel electrophoresis (SDS‐PAGE), the protein was electrotransferred onto a polyvinylidene fluoride membrane and blocked with 5% skim milk powder at room temperature for 1 hour. Then, the membrane was probed with diluted primary antibodies to GSK3β (1:5000, ab32391; Abcam), FTO (1:1500, ab126605; Abcam), MZF1 (1:500, ab64866; Abcam,), c‐Myc (1:1000, ab32072; Abcam), CDK2 (1:1000, ab32147; Abcam), CDK4 (1:500, ab137675; Abcam), Ki‐67 (1:1000, ab16667; Abcam), PCNA (1:1000, ab18197; Abcam), Bax (1:1000, ab199677; Abcam), Bcl‐2 (1:500, ab59348; Abcam) and glyceraldehyde‐3‐phosphate dehydrogenase (GAPDH) (ab9485, 1:2500; Abcam) at 4°C overnight. Next day, the membrane was re‐probed with horseradish peroxidase (HRP)‐labelled secondary antibody of goat anti‐rabbit antibody to immunoglobulin G (IgG) for 1 hour and visualized by enhanced chemiluminescence (ECL) kit (BB‐3501, Ameshame; Chiltem). Finally, Quantity One v4.6.2 software was used to quantify the grey levels of each band in the Western blot image. GAPDH served as an internal reference.

### 2.6 Co‐immunoprecipitation (Co‐IP) assay

Cells were lysed on ice using IP lysis buffer (Wuxi Biogoodland Biotechnology Co., Ltd.) supplemented with protease inhibitor. Then, 1 mg protein was taken from per sample and immunoprecipitated with monoclonal antibody to FTO for incubation overnight at 4℃. Next morning, 20 μL Protein A + G beads were added for 2 hours incubation. After that, the precipitates were eluted with IP lysis, and the sample was centrifuged at 2500 rpm for 5 minutes at 4℃ to discard the supernatant. Following addition of 20 μL 2 × Loading buffer into each well, the samples were subsequently subjected to SDS‐PAGE followed by Western blot analysis. The antibodies used were anti‐IgG (1:100, ab172730; Abcam), anti‐Ub (1:100, ab7780; Abcam) and anti‐FTO (1:50, # 31 687, CST).

### 2.7 Protein stability determination

Cyclohexanamide (CHX) treatment: To determine the FTO protein stability, the protein was extracted after centrifugation of CRC cells lysed with RIPA lysis buffer (P0013B; Beyotime) at 12 000 r/min and incubated with the protein synthesis inhibitor cyclohexanamide (CHX, 100 mg/mL) for 0 hour, 1 hour or 2 hours before analysis.

MG132 treatment: Cells were treated by 20 mM MG132 for 12 hours and then collected. Then, the cells were lysed in RIPA buffer containing 0.1% SDS and immunoblotted. ImageJ was used to quantify FTO expression normalized to GAPDH. The protein was extracted at 0 hour, 1 hour and 2 hours, and the expression of FTO was measured by Western blot analysis. The proteasome inhibitor MG132 (20 mM) and the CHX (100 mg/mL) were purchased from Sigma.

### 2.8 N6‐methyladenosine RNA binding protein immunoprecipitation (Me‐RIP)

Total RNA was isolated from CRC cells using the TRIzol method, in which mRNA was isolated and purified using PolyATtract® mRNA Isolation Systems (A‐Z5300; Aide Technology Co., Ltd.). Then, anti‐m6A antibody (1:500, ab151230; Abcam) or anti‐IgG antibody (ab109489, 1:100; Abcam) was added into IP buffer (20‐mM Tris pH 7.5, 140‐mM NaCl, 1% NP‐40, 2‐mM ethylenediaminetetraacetic acid (EDTA)) for 1 hour incubation with protein A/G magnetic beads. Next, isolated and purified mRNA and magnetic bead‐antibody complex were added into the IP buffer containing RNase inhibitor and protease inhibitor for incubation at 4°C overnight. The RNA was eluted, and MZF1 was analysed by RT‐qPCR after purification using phenol‐chloroform extraction.

### 2.9 Photoactivatable‐ribonucleoside‐enhanced cross‐linking and immunoprecipitation (PAR‐CLIP)

Colorectal carcinoma cells were cultured with 200 μM 4‐nitropyridine (Sigma) for 14 hours and cross‐linked at 365 nm with 0.4 J/cm^2^. Immunoprecipitation was carried out after lysis with FTO antibody overnight at 4°C. Then, the precipitated RNA was labelled with (γ‐32‐P)‐adenosine triphosphate (ATP) and visualized through autoradiography. For RT‐qPCR analysis, the precipitate was first detached with proteinase K to remove protein, and then, RNA extracted by TRIzol was used to perform RT‐qPCR to measure the expression of MZF1.

### 2.10 Cell counting kit‐8 (CCK‐8) method

The CRC cell proliferation was counted using the CCK‐8 (CK04, Dojindo, Kumamoto, Japan) method. Colorectal carcinoma cells in logarithmic growth were inoculated with 1 × 10^4^ cells/well in a 96‐well plate for 24 hours pre‐culture. After the transfection for 48 hours, 10 μL of CCK‐8 reagent was respectively added at 0 hour, 24 hours, 48 hours and 72 hours for 3 hours incubation at 37°C. The absorbance value of each well at wavelength of 450 nm was read using a microplate reader. The value was proportional to the number of cell proliferation in the culture medium, and the cell growth curve was plotted on this basis.

### 2.11 Flow cytometry

At 48 hours after transfection, cells were collected and detached with 0.25% trypsin to adjust the cell number to 1 × 10^6^ cells/mL followed by centrifugation to remove the supernatant. The cells were then fixed with addition of 70% pre‐chilled ethanol overnight at 4℃. Next day, the cells were washed twice with PBS, and 100 μL of the cell suspension was added with 50 μg of RNAase‐containing propidium iodide (PI) staining solution. After being protected from light for 30 min, the suspension was filtered through a 100‐mesh nylon mesh. A flow cytometer (BD, FL, NJ, USA) was used to assess the cell cycle. Then, the cells were resuspended with 200 μL binding buffer and stained with 10 μL Annexin V‐FITC (ab14085; Abcam) and 5 μL PI. After 15 minutes in the dark, the flow cytometer was employed to detect apoptosis.

### 2.12 Xenograft tumour in nude mice

Twenty‐four specific pathogen free female BALB/c nude mice (age: 6 weeks, weight: 15 ~ 18 g) were purchased from Slac Laboratory Animal Co., Ltd., and subcutaneously injected with SW620 cells stably transfected with oe‐NC, oe‐GSK3β + oe‐NC, or oe‐GSK3β + oe‐c‐Myc to establish a subcutaneous xenograft tumour model in nude mice. From the 7th day after inoculation, the tumour growth was observed every 3 days with the data recorded. Four weeks after inoculation, nude mice of each group were killed. The tumours were excised and weighed with a balance. The protein of the xenograft tumour tissues was extracted for Western blot analysis.

### 2.13 Statistical analysis

The SPSS 21.0 version (IBM Corp.) was used for statistical analysis. The measurement data were presented by mean ± standard deviation. The data of CRC tissues and adjacent normal tissues were analysed by paired *t* test, and the data of the other two groups were compared by unpaired Student's *t* test. The data among groups were analysed using one‐way analysis of variance (ANOVA) and Tukey's post hoc test, and the cell experimental data at different time points were compared using two‐way ANOVA. Tumour data at different time points were analysed by repeated measures ANOVA followed by Bonferroni's post hoc test. Statistical significance was assumed at *P* < .05.

## 3 RESULTS

### 3.1 GSK3β inhibited proliferation of CRC cells by down‐regulating FTO expression

**FIGURE 1:**
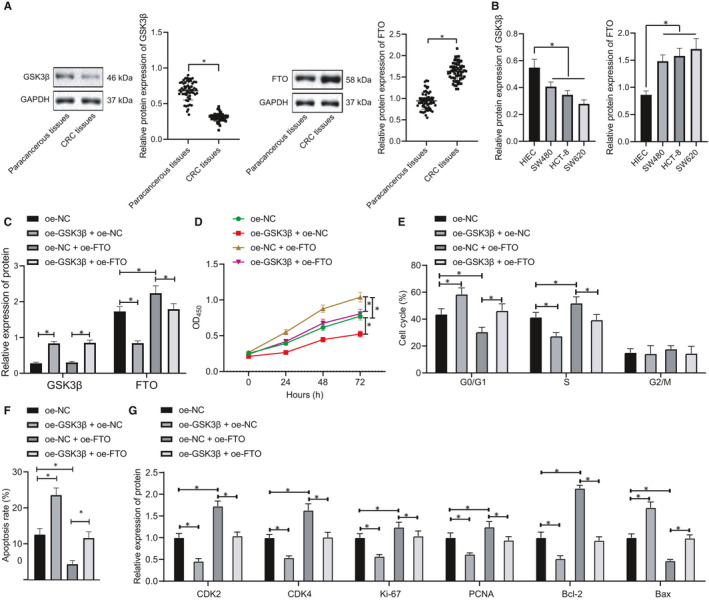
Kinase GSK3β represses CRC cell proliferation by reducing FTO expression. A, Western blot analysis of the expression of GSK3β and FTO in CRC tissues and adjacent normal tissues normalized to GAPDH, n = 57. B, Western blot analysis of the expression of GSK3β and FTO in CRC cells and immortalized normal HIECs. C, Western blot analysis of the expression of GSK3β and FTO in SW620 cells after oe‐GSK3β or oe‐FTO treatment normalized to GAPDH. D, The proliferative ability of SW620 cells in each group after oe‐GSK3β or oe‐FTO treatment assessed by CCK‐8 method. E, The cycle distribution of SW620 cells in each group after oe‐GSK3β or oe‐FTO treatment assessed by flow cytometry. F, The apoptotic capacity of SW620 cells in each group after oe‐GSK3β or oe‐FTO treatment assessed by flow cytometry. G, Western blot analysis of the expression of CDK2, CDK4, Ki‐67, PCNA, Bcl‐2 and Bax in SW620 cells after oe‐GSK3β or oe‐FTO treatment normalized to GAPDH. The data were expressed as mean ± standard deviation. The data of CRC tissues and adjacent normal tissues in normal distribution were analysed using paired *t* test, the data among groups were compared using one‐way ANOVA, and the data among multiple groups at different time were analysed by two‐way ANOVA. ^*^ *P* < .05

We collected clinical samples of CRC, cultured CRC cells (SW480, SW620 and HCT‐8 cells) and HIEC cells and performed Western blot and RT‐qPCR experiments to detect the expression of GSK3β and FTO in CRC tissues and cells. The results showed (Figure 1A,B) that the expression of GSK3β was significantly reduced in CRC tissues and cells, while the expression of FTO was significantly increased. Additionally, SW620 cells were used later due to the lowest GSK3β expression and the highest FTO expression among three CRC cells. In order to study whether GSK3β has an effect on the biological characteristics of CRC cells through mediating the expression of FTO, the SW620 cells were transfected with oe‐GSK3β or oe‐FTO. Western blot analysis showed (Figure 1C) overexpression of GSK3β significantly decreased the expression of FTO, but GSK3β was not affected by promoted FTO. After GSK3β was overexpressed, up‐regulating FTO could recover the expression of FTO but had no significant effect on GSK3β.

CCK‐8 assay was performed to detect the proliferation of SW620 and HCT‐8 cells in each group, and the results indicated (Figure 1D; Figure S1A) that overexpressing GSK3β noticeably reduced the cell proliferative potential, while increasing FTO expression led to a marked increase in SW620 cell proliferation. The inhibitory effect of GSK3β on the proliferation of SW620 cells could be reversed by overexpressing FTO. Flow cytometry results revealed (Figure 1E,F) that up‐regulating GSK3β could cause a significant increase in the percentage of G0‐/G1‐phase cells and a decrease in the amount of S‐phase cells, leading to increased proportion of apoptotic cells. By contrast, overexpression of FTO could significantly decline the percentage of G0‐/G1‐phase cells while increasing the quantity of S‐phase cells, resulting in decreased proportion of apoptotic cells. The promotive effect of GSK3β on apoptosis could be rescued by promoting FTO expression. Western blot analysis for determining the expression of proteins related to proliferation, apoptosis and cycle of each group of SW620 cells uncovered that (Figure 1G) up‐regulating GSK3β significantly reduced the expression of CDK2, CDK4, Ki‐67, PCNA and Bcl‐2 while promoting Bax expression. Overexpression of FTO noticeably facilitated the expression of CDK2, CDK4, Ki‐67, PCNA and Bcl‐2 while attenuating Bax expression. The regulatory role of GSK3β in the expression of the above proteins could be restored by increasing FTO expression. The above results supported that GSK3β can inhibit the proliferation of SW620 cells by down‐regulating the expression of FTO.

### 3.2 GSK3β mediated the stability of m6A demethylase FTO in CRC cells

**FIGURE 2:**
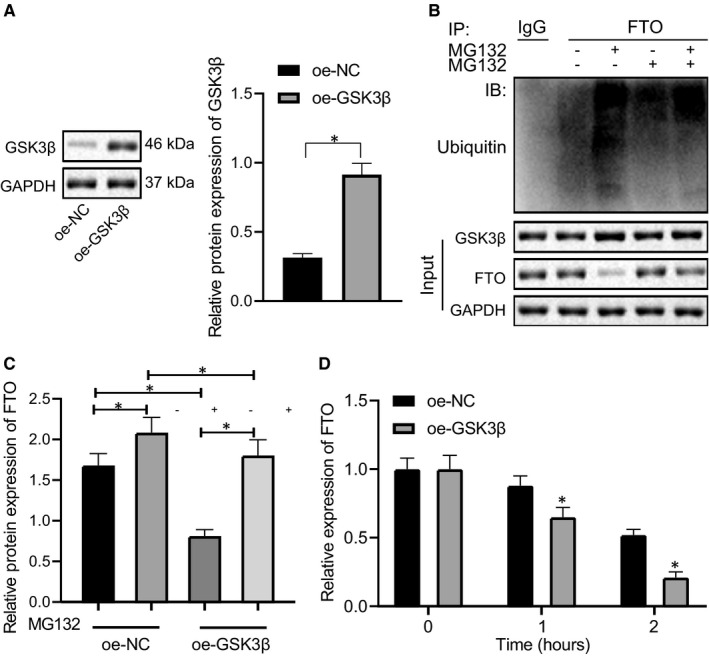
GSK3β inhibits the expression of m6A demethylase FTO in CRC cells. A, Western blot analysis of GSK3β expression after oe‐GSK3β treatment. B, Co‐IP detection of FTO ubiquitination in SW620 cells. C, Western blot analysis of FTO protein expression after treatment of oe‐GSK3β combined with proteasome inhibitor MG132. D, Western blot analysis of FTO protein stability after treatment of oe‐GSK3β combined with CHX. GAPDH was used as the internal reference. The data were expressed as mean ± standard deviation. The data of CRC tissues and adjacent normal tissues in normal distribution were analysed using paired *t* test, the data among groups were compared using one‐way ANOVA, and the data among multiple groups at different time were analysed by two‐way ANOVA. ^*^ *P* < .05

From the above results, we know that the kinase GSK3β could affect the proliferation, apoptosis and cycle distribution of CRC cells by mediating the protein level of FTO. Additionally, studies in existing literature have shown that GSK3β could promote FTO ubiquitination by mediating FTO phosphorylation, thereby promoting FTO degradation and reducing FTO protein expression.^8^ In order to further investigate whether GSK3β could mediate the ubiquitination of FTO in CRC, GSK3β was overexpressed in CRC cells. Western blot analysis results revealed that (Figure 2A) the expression of GSK3β significantly increased after overexpression of GSK3β. The Co‐IP test for detecting the binding of FTO to ubiquitin in CRC cells indicated (Figure 2B) that the binding amount of FTO to ubiquitin increased after overexpressing GSK3β, that is, the ubiquitination of FTO increased. After treatment with the proteasome inhibitor MG132 (Figure 2C), the ubiquitination degradation of FTO was inhibited, and after increasing GSK3β expression, FTO was degraded due to a high degree of ubiquitination, exhibiting a decline in the expression of FTO protein. The results of CHX treatment for detecting the stability of FTO protein showed that (Figure 2D) overexpression of GSK3β significantly reduced the protein stability of FTO. Altogether, the kinase GSK3β can mediate the ubiquitination of m6A demethylase FTO to inhibit FTO expression in CRC cells.

### 3.3 GSK3β inhibited MZF1 expression by mediating FTO‐mediated m6A modification of MZF1 in CRC cells

**FIGURE 3:**
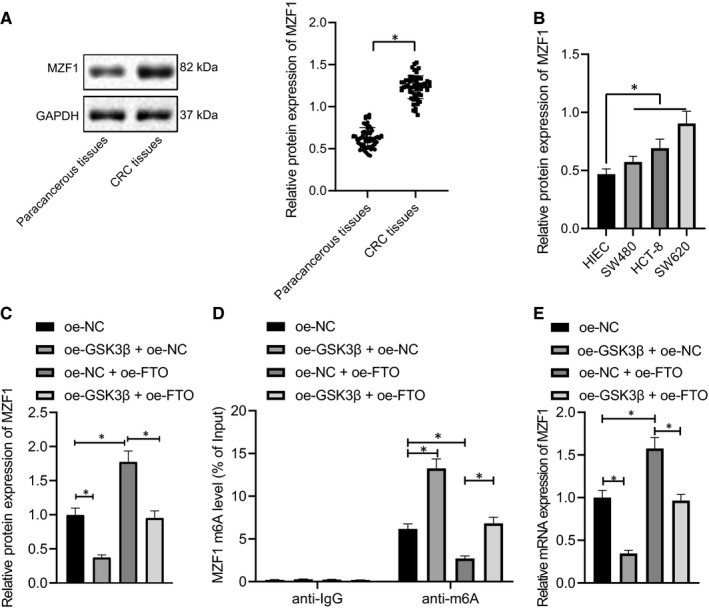
GSK3β decreases MZF1 expression by regulating FTO‐mediated m6A modification of MZF1. A, Western blot analysis of the expression of MZF1 in CRC tissues and adjacent normal tissues, n = 45. B, Western blot analysis of the expression of MZF1 in SW480, SW620 and HCT‐8 and immortalized normal HIECs. C, Western blot analysis of the expression of MZF1 in SW620 cells after oe‐GSK3β or oe‐FTO treatment. D, m6A modification level of MZF1 in SW620 cells after oe‐GSK3β or oe‐FTO treatment assessed by Me‐RIP. E, The binding of FTO to MZF1 mRNA after oe‐GSK3β or oe‐FTO treatment assessed by PAR‐CLIP. GAPDH was applied as the internal reference. The data were expressed as mean ± standard deviation. The data of CRC tissues and adjacent normal tissues in normal distribution were analysed using paired *t* test, the data among groups were compared using one‐way ANOVA, and the data among multiple groups at different time were analysed by two‐way ANOVA. ^*^ *P* < .05

Previous study has shown that FTO promotes MZF1 expression by removing m6A mRNA modification of MZF1.^11^ First, the expression of MZF1 in CRC tissues and cells was assessed by Western blot analysis, and the results showed (Figure 3A,B) that MZF1 expression was significantly higher in CRC tissues and cells. Next, the Western blot analysis showed (Figure 3C, Figure S1B) that the expression of MZF1 was significantly reduced after overexpressing GSK3β and was markedly increased after up‐regulating FTO expression in SW620 and HCT‐8 cells. The restraining effect of GSK3β on MZF1 could be reversed by overexpression of FTO in SW620 cells. Me‐RIP for examining the m6A modification level of MZF1 in each group displayed (Figure 3D; Figure S1C) that the m6A modification level of MZF1 was significantly increased after highly expressing GSK3β, but was noticeably diminished after overexpressing FTO. The promotive role of GSK3β on m6A modification level of MZF1 could be rescued by high expression of FTO. The results of PAR‐CLIP experiment for detection of the binding of FTO to MZF1 mRNA uncovered (Figure 3E; Figure S1D) that the binding amount of FTO to MZF1 mRNA was significantly inhibited after stimulating GSK3β expression, but was attenuated after increasing FTO expression. The inhibitory effect of GSK3β on the binding of FTO to MZF1 mRNA could be restored by overexpressing FTO. Taken together, the kinase GSK3β inhibited MZF1 expression by regulating FTO‐mediated m6A modification of MZF1.

### 3.4 FTO reduced c‐Myc expression by down‐regulating MZF1

**FIGURE 4:**
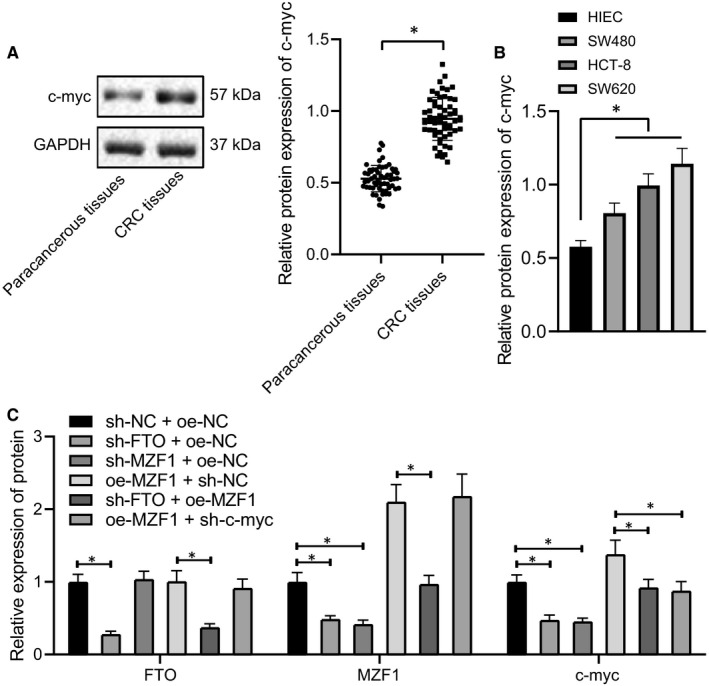
FTO impedes c‐Myc expression by down‐regulating MZF1. A, Western blot analysis of the expression of c‐Myc in CRC tissues and adjacent normal tissues, n = 45. B, Western blot analysis of the expression of c‐Myc in CRC cells and immortalized normal HIECs. C, Western blot analysis of the expression of FTO, MZF1, and c‐Myc in SW620 cells after sh‐FTO, oe‐MZF1 or sh‐c‐Myc treatment. GAPDH was employed as the internal reference. The data were expressed as mean ± standard deviation. The data of CRC tissues and adjacent normal tissues in normal distribution were analysed using paired *t* test, the data among groups were compared using one‐way ANOVA, and the data among multiple groups at different time were analysed by two‐way ANOVA. ^*^ *P* < .05

Existing literature has shown that MZF1 could promote the expression of the proto‐oncogene c‐Myc,^14^ and c‐Myc could contribute to the occurrence of CRC.^15^ Western blot analysis determining the expression of c‐Myc in CRC tissues and cells revealed that (Figure 4A,B) the expression of c‐Myc was significantly increased in CRC tissues and cells. Next, the expression of FTO, MZF1 and c‐Myc in each group of cells was assessed by Western blot analysis. The results showed (Figure 4C) that low expression of FTO led to a significant decrease in the expression of MZF1 and c‐Myc in SW620 cells, and high expression of MZF1 exerted no obvious effect on FTO expression but led to increased c‐Myc expression. After under‐expressing FTO, up‐regulating MZF1 significantly promoted the expression of MZF1 and c‐Myc while not affecting FTO expression. After overexpressing MZF1, down‐modulating c‐Myc caused no significant changes in FTO and MZF1 expression while reducing the c‐Myc expression. Additionally, the expression levels of MZF1 and c‐Myc in sh‐MZF1 + oe‐NC group were significantly lower than those in sh‐NC + oe‐NC group, indicating that FTO mediated the expression of c‐Myc by regulating MZF1. Collectively, FTO inhibited c‐Myc expression through negatively modulating MZF1.

### 3.5 FTO activated MZF1/c‐Myc axis to promote CRC cell proliferation

**FIGURE 5:**
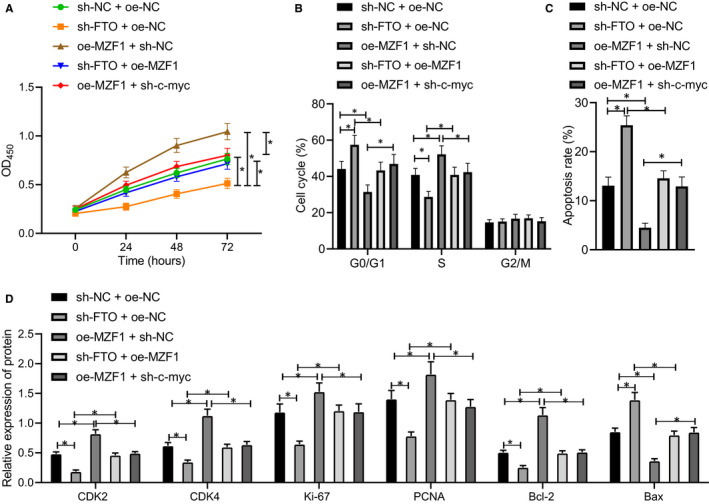
FTO up‐regulates MZF1/c‐Myc axis to induce CRC cell proliferation. A, CCK‐8 method determining the SW620 cell proliferative ability after sh‐FTO, oe‐MZF1 or sh‐c‐Myc treatment. B, Flow cytometry assessing the cycle distribution of SW620 cells after sh‐FTO, oe‐MZF1, or sh‐c‐Myc treatment. C, Flow cytometry examining the apoptosis of SW620 cells after sh‐FTO, oe‐MZF1 or sh‐c‐Myc treatment. D, Western blot analysis of the expression of CDK2, CDK4, Ki‐67, PCNA, Bcl‐2, and Bax genes in SW620 cells after sh‐FTO, oe‐MZF1 or sh‐c‐Myc treatment normalized to GAPDH. The data were expressed as mean ± standard deviation. The data among groups were compared using one‐way ANOVA, and the data among multiple groups at different time were analysed by two‐way ANOVA. ^*^ *P* < .05

Furthermore, the functions of FTO in SW620 cell biological processes were analysed with the involvement of MZF1/c‐Myc axis. The results of CCK‐8 for detecting the proliferation of SW620 and HCT‐8 cells revealed (Figure 5A; Figure S1E) that low expression of FTO noticeably reduced the proliferative potential of SW620 cells, while overexpression of MZF1 increased the cell proliferation. The inhibitory effect of under‐expressed FTO on SW620 cell proliferative ability could be restored by up‐regulating MZF1, and the promotive role of overexpressed MZF1 in SW620 cell proliferation could be reversed by down‐regulation of c‐Myc. Flow cytometry was used to detect cycle distribution and apoptosis of SW620 cells in each group, and the results indicated (Figure 5B,C) that under‐expressed FTO significantly increased the ratio of G0‐/G1‐phase cells, decreased the proportion of S‐phase cells and promoted SW620 cell apoptosis. Overexpressed MZF1 noticeably inhibited the ratio of G0‐/G1‐phase cells, promoted the proportion of S‐phase cells and suppressed SW620 cell apoptosis. The promotive effect of under‐expressed FTO on apoptosis could be reversed by overexpression of MZF1, and negative modulation of c‐Myc could restore the inhibitory effect of overexpressed MZF1 on apoptosis. The results of Western blot analysis for assessing expression of proliferation‐, apoptosis‐ and cycle‐related proteins in cells of each group uncovered (Figure 5D) that down‐regulated FTO significantly reduced the expression of CDK2, CDK4, Ki‐67, PCNA and Bcl‐2 while increasing Bax expression, and overexpressing MZF1 noticeably increased the expression of CDK2, CDK4, Ki‐67, PCNA and Bcl‐2 while suppressing Bax expression. The regulatory effect of down‐regulated FTO on the above proteins could be recovered by overexpressing MZF1. After overexpressing MZF1, down‐regulating c‐Myc decreased the expression of CDK2, CDK4, Ki‐67, PCNA and Bcl‐2 and increased the Bax expression. Collectively, FTO promoted the proliferation of SW620 cells by up‐regulating MZF1/c‐Myc axis.

### 3.6 GSK3β inhibited the growth of CRC xenografts by suppressing the expression of c‐Myc

**FIGURE 6:**
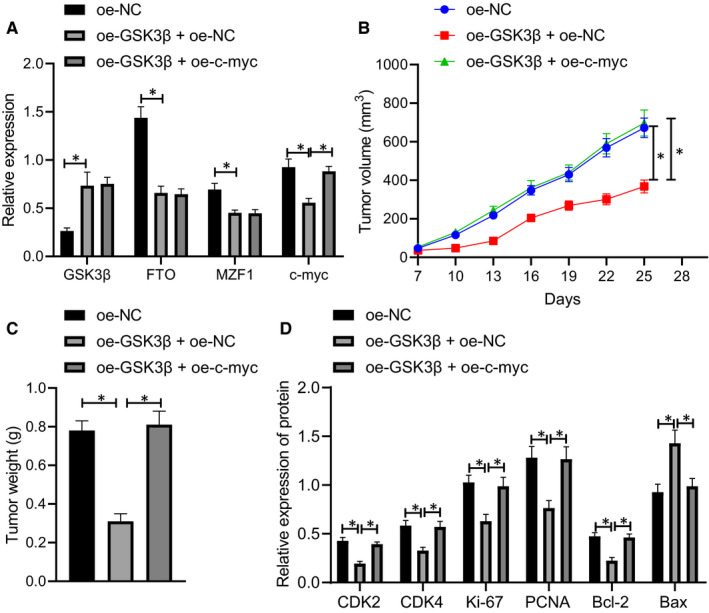
GSK3β inhibits the growth of CRC xenografts by reducing the expression of c‐Myc. A, Western blot analysis of the expression of GSK3β, FTO, MZF1 and c‐Myc in nude mice bearing cells expressing oe‐GSK3β or oe‐c‐Myc, normalized to GAPDH. B, Volume of xenografts in nude mice bearing cells expressing oe‐GSK3β or oe‐c‐Myc. C, Weight of xenografts in nude mice bearing cells expressing oe‐GSK3β or oe‐c‐Myc. D, Western blot analysis of the expression of CDK2, CDK4, Ki‐67, PCNA, Bcl‐2 and Bax in xenografts in nude mice bearing cells expressing oe‐GSK3β or oe‐c‐Myc, normalized to GAPDH. The data were expressed as mean ± standard deviation. The data among groups were compared using one‐way ANOVA, and the data among multiple groups at different time were analysed by two‐way ANOVA, n = 8. ^*^ *P* < .05

In order to further study the effect of GSK3β on the growth of CRC xenograft tumours in vivo by mediating the expression of c‐Myc, the stably transfected SW620 cell lines were screened, and the stably transfected SW620 cell suspension was injected subcutaneously to establish the subcutaneous xenograft model of nude mice. The results of Western blot experiment showed (Figure 6A) that overexpressing GSK3β significantly reduced the expression of FTO, MZF1 and c‐Myc, and then up‐regulating c‐Myc caused no changes in the expression of GSK3β, FTO and MZF1 while recovering the c‐Myc expression.

Observation results of tumour formation in nude mice indicated (Figure 6B,C) that overexpressed GSK3β significantly reduced tumour volume and weight, which could be restored by up‐regulating c‐Myc expression. Western blot analysis of protein extracted from xenograft tissues indicated (Figure 6D) that overexpressed GSK3β noticeably reduced the expression of CDK2, CDK4, Ki‐67, PCNA and Bcl‐2 while increasing Bax expression, which could be recovered by positive modulation of c‐Myc. The above research results unravelled that the kinase GSK3β could inhibit the growth of CRC xenografts by suppressing the expression of c‐Myc.

## 4 DISCUSSION

**FIGURE 7:**
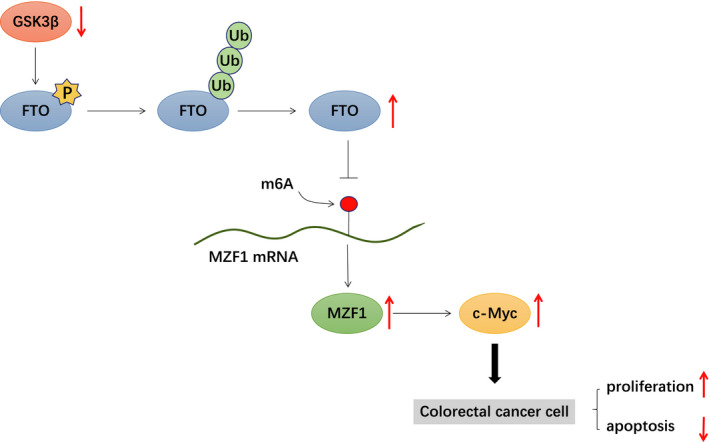
Schematic map concerning the role of GSK3β in CRC. GSK3β could suppress the initiation and progression of CRC through FTO‐mediated MZF1/c‐Myc axis

Colorectal carcinoma ranks second among the list of the most common cancers diagnosed in female and third in male, accounting for about 10% of total cancer cases diagnosed annually and leading to numerous cancer‐related deaths.^4^ Furthermore, the incidence of this malignant tumour is rapidly increasing in developing countries like China who are facing development in economy and variations in daily food and lifestyle,^1^ which urges the scientists to find new effective therapeutic agents for improving the dismal prognosis of patients with CRC. The kinase GSK3β has been reported as a regulator in the PI3K pathway that is associated with the progression of CRC,^5^, ^6^ but the underlying role of GSK3β in CRC remains veiled. On this basis, we proved the hypothesis that GSK3β could suppress the initiation and progression of CRC through FTO‐mediated MZF1/c‐Myc axis (Figure 7).

Initially, we determined that could GSK3β suppress the expression of FTO by mediating the ubiquitination of FTO and further found that GSK3β has a substantial effect on the cycle, proliferative potential and apoptosis of CRC cells by regulating FTO expression. Mounting evidence shows that GSK3β modulates signal transduction pathways including protein kinase and insulin signalling in a PI3K‐dependent manner, and it can phosphorylate FTO to cause polyubiquitination, leading to a decline in FTO expression,^8^ as confirmed by the Co‐IP experiment in this paper. Besides, GSK3β is known as a very important regulatory protein to modulate the level of cyclin D1,^7^ thus playing a vital role in cell cycle, proliferation and apoptosis. It is also reported that abnormalities of m6A modification and the related proteins such as FTO contribute to the initiation, development and cell viability of various cancers like liver and cervical cancer,^16^ which is partly in line with the above finding.

In the subsequent experiments, we found highly expressed MZF1 and c‐Myc in CRC and that FTO could activate the MZF1/c‐Myc axis to promote the proliferative ability of CRC cells. Existing literature has shown that depletion of FTO effectively suppressed the proliferative and invasive potential of cells, and FTO increased MZF1 expression by demethylating MZF1 mRNA to play its oncogenic role in lung cancer.^11^ MZF1 is reported to function as a tumour promoter and is expected to facilitate cancer development, such as colorectal, lung and liver cancers.^12^ Additionally, MZF1 could positively modulate the expression of c‐Myc, and MZF1‐regulated c‐Myc expression may enhance the progression of lung adenocarcinoma.^14^ The proto‐oncogene c‐Myc has been determined as an important participator in cell growth and as a primary human oncogene when expressed in an unproper fashion.^17^ Wang et al also reported that c‐Myc could lead to an increase in cell growth in CRC and promote CRC initiation.^18^ Thus, it is essential to normalize the expression of c‐Myc in cells to avoid the occurrence of malignant tumours.^19^

Furthermore, our findings indicated that GSK3β could impede the expression of c‐Myc to inhibit the growth of CRC xenografts in nude mice, providing in vivo evidence that GSK3β has an inhibitory effect on the development of CRC. As reported by Huang *et al*, GSK3β could restrain β‐catenin and its downstream targets including c‐Myc to hamper cancer progression.^20^ Likewise, zinc finger protein 746 aggravates the symptoms of CRC *via* GSK3β‐mediated c‐Myc stability,^21^ further confirming the role of GSK3β and c‐Myc in CRC. Collectively, GSK3β could reduce the expression of MZF1 through restraining FTO expression and thus lead to a decrease in the c‐MYC expression, thus inhibiting the proliferative and migratory phenotypes of CRC cells while promoting their apoptosis.

## 5 CONCLUSIONS

In conclusion, this study finds a novel GSK3β/FTO/MZF1/c‐MYC axis which is involved in the occurrence and development of CRC and, clinically, proposes a therapeutic regimen based on using GSK3β as the inhibitor for treating CRC patients, which may hold great potential to relieve CRC‐related pathogenic symptoms. In the future, we will study in more detail the role of GSK3β on CRC through FTO‐regulated MZF1/c‐MYC axis by adding different cell lines used in experiments, etc, and seek to find more effective therapies for treating this disease as the next action.

## CONFLICT OF INTEREST

All the authors declare that they have no competing interests.

## AUTHOR CONTRIBUTIONS

**Zeyan Zhang:** Conceptualization (lead); Investigation (lead); Writing‐original draft (lead). **Qianfu Gao:** Methodology (equal); Resources (equal); Supervision (equal). **Shanchao Wang:** Data curation (equal); Formal analysis (equal); Project administration (equal); Writing‐review & editing (equal).

## Supporting information

Fig S1

## Data Availability

Research data are not shared.
